# The safety of morphine in patients with acute heart failure: A systematic review and meta‐analysis

**DOI:** 10.1002/clc.23691

**Published:** 2021-07-08

**Authors:** Dandan Zhang, Wei Lai, Xiao Liu, Yang Shen, Kui Hong

**Affiliations:** ^1^ Cardiology Department The Second Affiliated Hospital of Nanchang University Nanchang Jiangxi China; ^2^ Department of Genetic Medicine The Second Affiliated Hospital of Nanchang University Nanchang Jiangxi China; ^3^ Jiangxi Key Laboratory of Molecular Medicine Nanchang Jiangxi China

**Keywords:** acute heart failure, all‐cause mortality, in‐hospital mortality, invasive ventilation, morphine

## Abstract

While morphine has long been widely used in treating acute heart failure (AHF) due to its vasodilatory properties and anticipated anxiolysis, it remains unclear whether the application of morphine to those patients is reasonable. We aim to conduct a systematic review and meta‐analysis to assess the safety of morphine in patients with AHF. We searched PubMed, Cochrane Library, and Embase electronic databases from inception through March 2020. Pooled odds ratios (ORs) and 95% confidence intervals (CIs) were used to estimate the outcomes. Seven studies with 172, 226 patients were included. The results showed that morphine usage was not associated with increased in‐hospital mortality (OR: 1.94; 95% CI 0.93 to 4.03; *p* = 0.08). However, the use of morphine significantly increased the risk of invasive ventilation (OR: 2.72; 95% CI 1.09 to 6.80; *p* = 0.03). Furthermore, the subgroup analysis indicated that the application of morphine was not associated with increased 7‐day all‐cause mortality in patients with AHF (OR: 1.69; 95% CI 0.80 to 3.22; *p* = 0.11) but significantly increased the risk of 30‐day all‐cause mortality (OR: 1.59; 95% CI 1.16 to 2.17; *p* = 0.004). Based on current evidence, our results suggested that although morphine therapy did not significantly increase the risk of short‐term death (in the hospital or within 7 days) in patients with AHF, the risk of long‐term death and invasive ventilation were significantly increased. This result needs to be further confirmed by an ongoing randomized control trial.

## 1 INTRODUCTION

Acute heart failure (AHF) is a rapidly increasing worldwide health problem with a high mortality rate and a high economic burden that has a high impact on health systems. It is broadly defined as a rapid onset of new or worsening symptoms of heart failure, which itself is not a single disease entity but rather a syndrome of the worsening of signs and symptoms reflecting an inability of the heart to pump blood at a rate commensurate to the needs of the body,^1^ requiring urgent hospital admission for evaluation and treatment.^2^

Morphine, first isolated in the early 1800s,^3^ has been used in patients with AHF due to its anticipated anxiolytic and vasodilatory properties for many years^15^ However, to date, the clinical effects of using morphine in patients with AHF remain controversial. It seems that the results from several retrospective studies do not support the safety of morphine in the treatment of patients with AHF.^4^, ^5^ The collected evidence suggested that applying morphine to patients with AHF may increase adverse events, which included a greater frequency of mechanical ventilation, prolonged hospitalization, more ICU admissions, and increased in‐hospital mortality. However, other studies did not find this association.^6^, ^7^ Thereafter, several articles have reviewed published studies but have not given clear support or negative answers about the effects of morphine on patients with AHF.^8^, ^9^, ^10^

Despite increasing interest in the field of palliative care for heart failure, to date, there has been no clear uniform standard for the use of morphine. The 2017 Association/American College of Cardiology Heart Failure guidelines^11^ do not mention morphine either in the routine treatment of AHF or in late palliative care. In addition, the European Society of Cardiology^12^ does not recommend routine use, only for refractory dyspnea as palliative care in advanced HF patients, and suggests that morphine be cautiously considered in patients with severe dyspnea, mostly with pulmonary edema (class IIb recommendation; the level of evidence B) due to side effects from its use, including nausea, hypotension, bradycardia, and respiratory depression (potentially increasing the need for invasive ventilation) in patients with AHF. Thereafter, several articles^13^, ^14^ advised against the use of morphine to treat patients with AHF. However, to the best of our knowledge, despite a certain amount of data in the literature, there are no meta‐analysis studies that have evaluated the real effects of morphine on patients with AHF.

More importantly, in addition to the anticipated anxiolytic and vasodilatory properties, morphine also showed significant side effects, such as vomiting and aspiration, which may increase mortality.^15^, ^16^ Although respiratory depressive effects can be reversed by an appropriate dose of naloxone, morphine‐induced hypotension has the potential to decrease myocardial perfusion and increase myocardial ischemia, ultimately resulting in death from cardiogenic shock. It is important, therefore, to comprehensively evaluate the effect of morphine in the treatment of heart failure.^17^, ^18^ Therefore, to fully evaluate the benefits and risks in the absence of evidence from any randomized controlled trial (RTC), we conducted a comprehensive meta‐analysis of all available studies to elucidate the effect of morphine on patients with AHF.

## 2 METHODS

### 2.1 Literature search

This meta‐analysis was conducted according to the Preferred Reporting Items for Systematic Reviews and Meta‐Analysis guidelines.^19^ We systematically conducted a computerized search through the PubMed, Cochrane Library, and EMBASE databases for eligible studies up to March 2020. No geographic restriction was applied in the search process. A comprehensive search strategy was developed based on the following terms: (1) Morphine and intravenous morphine and (2) AHF, acute decompensated heart failure, and acute pulmonary edema. In addition, they were combined with Boolean operators “*AND*”.

Studies were taken into account when they satisfied the following inclusion criteria: (1) Patients treated with morphine as the case group and patients without morphine as the control group; (2) Contained extractable data, such as all‐cause mortality, in‐hospital mortality, and the risk of invasive ventilation. Exclusion criteria were as follows: (1) Studies not pertinent to morphine or AHF; (2) Lack of a control group; (3) Publication with insufficient data (e.g., abstract, editorial, and comment); and (4) Non‐English studies.

### 2.2 Data extraction and quality assessment

All papers were independently screened by two reviewers (Dandan Zhang and Wei Lai) according to the search strategy. The first phase of screening was performed by reading abstracts, and the second phase of screening involved reviewing the full text. Ultimately, articles meeting the eligibility criteria were further reviewed. Extracted databases were then cross‐checked between the two authors to rule out any discrepancies. Additionally, disagreement was resolved by discussion with a third investigator (Kui Hong). If both unadjusted and adjusted ORs existed in one study, we extracted the most completely adjusted ORs. From each study, extracted information included the following elements: name of the first author, year of publication, country, study design, inclusion criteria, total number of patients, proportion of male patients, age, all‐cause mortality, in‐hospital mortality, and invasive mechanical ventilation. All studies were evaluated using the Newcastle‐Ottawa Scale (NOS) for observational studies. The validated NOS items with a total of nine stars involved three terms, including the selection of the population, the comparability of the study, and the assessment of the outcome.

### 2.3 Statistical analysis

All statistical analyses were performed by using RevMan Manager version 5.3 (The Cochrane Collaboration 2014; Nordic Cochrane Center Copenhagen, Denmark). We used the odds ratio (OR) with 95% confidence intervals (CIs) to evaluate the endpoints. We used the Newcastle‐Ottawa quality assessment scale (NOS) to evaluate the quality of all included studies.^20^ A study with a NOS score of ≥6 stars was regarded as high‐quality; otherwise, it was regarded as a low‐quality study.^21^, ^22^

The choice between fixed or random effects models was determined by evaluating the heterogeneity as recommended by the scientific statement of the American Heart Association. The between‐study heterogeneity was assessed quantitatively using the I^2^ test. I^2^ values of 25%, 50%, and 75% were considered to represent low, moderate, and large heterogeneity, respectively. When significant heterogeneity existed across the included studies, a random effects model was used for the analysis. If this was not the case, the fixed‐effect model was used. The statistical significance threshold was set at *p* < 0.05.

## 3 RESULTS

### 3.1 Study selection

**FIGURE 1:**
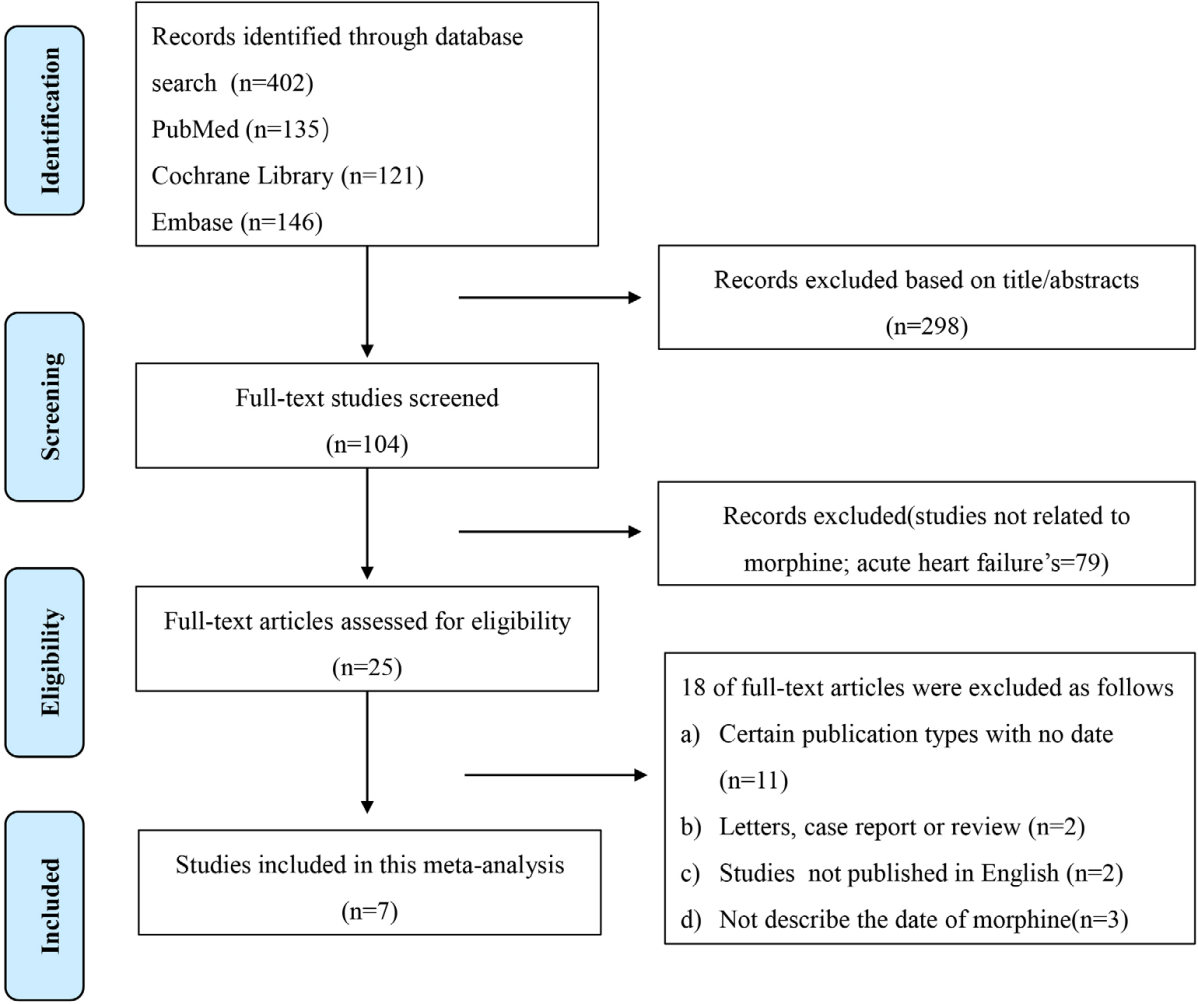
Flowchart of the study selection process for the meta‐analysis

Following the above search strategies, a total of 402 articles were screened out in the initial database search: 135 of PubMed, 146 of EMBASE, and 121 of the Cochrane Library. After excluding duplicates and screening the titles and abstracts according to the inclusion and exclusion criteria, 25 studies were reviewed. Eighteen records were excluded for other reasons. Finally, seven studies met the inclusion criteria and were included in the analysis. The flow diagram of the search steps is illustrated in Figure 1.

### 3.2 Characteristics of included studies and quality assessment

Seven studies with 22 967 cases and 172 226 patients with AHF were included in this meta‐analysis.^4^, ^5^, ^6^, ^7^, ^14^, ^23^, ^24^ Overall, these seven studies were published from 1999 to 2019 and emerged from different countries, including Spain,^14^, ^23^ USA,^4^, ^5^ Israel,^6^, ^24^ and UK.^7^ The sample sizes of the included studies varied from 181 to 147 362. The mean age ranged from 73 to 81 years. The duration of follow‐up across the studies varied from 48 h to 30 days. Among the seven studies, most were retrospective case–control studies. The detailed characteristics of the selected studies are presented in Table 1. The methodological quality of the included studies was considered to have a low risk of bias, with 6–7 stars (Table S1).

**TABLE 1:** Basic characteristics of seven articles included in the meta‐analysis

First Author/ Publication year	Country	Study design	Patients source/Period	Number of patients: (Morphine/Control group)	Age, mean ± *SD*: (Morphine/Control group)	Male, %: (Morphine/Control group)	Outcomes assessed	Comorbidities, % (Morphine/Control group)	Adjustment for confounders
Sachetti et al. 1999^4^	USA	Retro	OLLMCEM， 1992 ~ 1996	88/93	NA	NA	NIMV; Number of patients admitted to the intensive care unit	NR	Age, diagnoses, prehospital and ED pharmacological interventions
Peacock et al. 2010^5^	USA	Retro	ADHERE, (…) ~ 2004	20 782/126580	73.2 ± 15.3/ 75.4 ± 13.8	47/ 48	NIMV; In‐hospital mortality; Number of patients admitted to the intensive care unit	Atrial fibrillation: 27.5/31.5 Prior myocardial infarction: 38.7/34.4 Chronic dialysis: 6.1/4.4 COPD/asthma: 33.5/31.0 Diabetes: 44.9/44.0 Hypertension: 75.2/73.2 Stroke: 17.3/16.7	Age, BUN, systolic blood pressure, creatinine, dyspnea at rest, chronic dialysis, heart rate, and abnormal troponin
Gray et al. 2010^7^	UK	Retro	3CPO trial, 2003 ~ 2007	541/511	77.6 ± 9.6/ 77.7 ± 9.8	45/ 43	7‐day mortality Change from baseline dyspnea	Cerebrovascular disease: 16.8/16.6 COPD/asthma: 17.0/18.4 Diabetes: 29.4/30.3 Hypertension: 51.9/54.7 Ischemic heart disease: 66.4/61.9 Valvular heart disease: 11.1/10.8	Age, ability to obey commands, and baseline systolic blood pressure
Iakobishviliz et al. 2011 ^6^	Israel	Retro	HFSIS, 2003 ~ 2003	218/2118	76.0 ± 12.6/ 76.0 ± 11.1	48/ 44	In‐hospital mortality; 30‐day mortality	Atrial fibrillation: 26.1/29.4 Acute coronary syndrome: 65.6/36.8 COPD/asthma:19.3/19.3 Dyslipidemia: 65.6/57.4 Diabetes: 60.1/50.8 Hypertension: 79.4/76.1 Stroke: 13.8/11.4 Prior myocardial infarction: 37.2/37.2	Admission heart rate, serum urea, serum creatinine, glucose; white blood cell count, systolic blood pressure, Killip class on admission, dyslipidemia, acute coronary syndrome, and chronic treatment with furosemide, the use of intravenous inotropes and vasodilators
Oscar et al. 2017^14^	Spain	Retro	EAHFE, 2011 ~ 2014	416/6100	80.7 ± 10.2/ 81.1 ± 10.1	57/ 57	NIMV; In‐hospital mortality; 7‐day mortality; 30‐day mortality	Atrial fibrillation: 44.7/39.3 COPD/asthma: 21.8/21.1 Diabetes: 45.8/50.9 Dyslipidemia: 39.3/46.9 Hypertension: 84.0/90.9 Ischemic heart disease: 36.4/37.1 Valvular heart disease: 25.8/27.6	Ischemic heart disease, cerebrovascular disease, atrial fibrillation, peripheral vascular disease, chronic obstructive pulmonary disease, dementia, Barthel index (points), cardio‐respiratory (NYHA III‐IV), loop‐diuretics, nitrates, antiplatelets, anticoagulants; systolic blood pressure, heart rate, basal oxygen saturation, atrial fibrillation, left ventricular hypertrophy, left or right bundle branch block, glucose, creatinine clearance, potassium, intravenous nitrates, need for vasoactive/ inotropes drugs, non‐invasive ventilation, any kind of ventilatory support
Dominguez et al. 2017 ^23^	Spain	Retro	HES, 2013 ~ 2015	161/830	NR	NA	In‐hospital mortality	NR	Age, sex, previous episode of HF, chronic kidney disease, chronic use of beta‐blockers and diuretics, and LVEF < 50%.
Oren et al. 2019^24^	Israel	Retro	RMCHI, 2005 ~ 2016	761/13027	78.0 ± 11.0/ 75.0 ± 12.0	42/ 50	NIMV; In‐hospital mortality	Atrial fibrillation: 41.1/41.8 COPD/asthma: 14.7/13.4 Diabetes: 54.9/53.7 Hypertension: 75.6/75.1 Valvular heart disease: 10.0/11.0 Prior myocardial infarction: 28.1/ 26.3	Age, male, hypertension, diabetes, prior myocardial infarction, chronic lung disease, AF, valvular heart disease, systolic blood pressure, heart rate, serum creatinine, BUN, glucose, baseline hemoglobin, white blood cell count, background medical therapy antiplatelet agents, beta‐blockers, ACEI /ARB, furosemide, metolazone, anticoagulants

Abbreviations: ACEI, angiotensin‐converting enzyme inhibitors; ADHERE, acute decompensated heart failure national registry; AF, atrial fibrillation; ARB, angiotensin‐II receptor blockers; BUN, blood urea nitrogen; COPD, chronic obstructive pulmonary disease; CPO, cardiogenic pulmonary edema; ED, emergency department; HES, hospital emergency services; HF, heart failure; HFSIS, heart failure survey in Israel; LVEF, left ventricular ejection fraction; MIMO, The Midazolam versus Morphine in APE trial; NA, not available; NIMV, need for invasive mechanical ventilation; OLLMCEM, acute pulmonary edema from “Our Lady of Lourdes Medical Center of Emergency Medicine”; Retro, retrospective cohort; RMCHI, Rambam Medical Center, Haifa, Israel.

### 3.3 In‐hospital mortality

**FIGURE 2:**
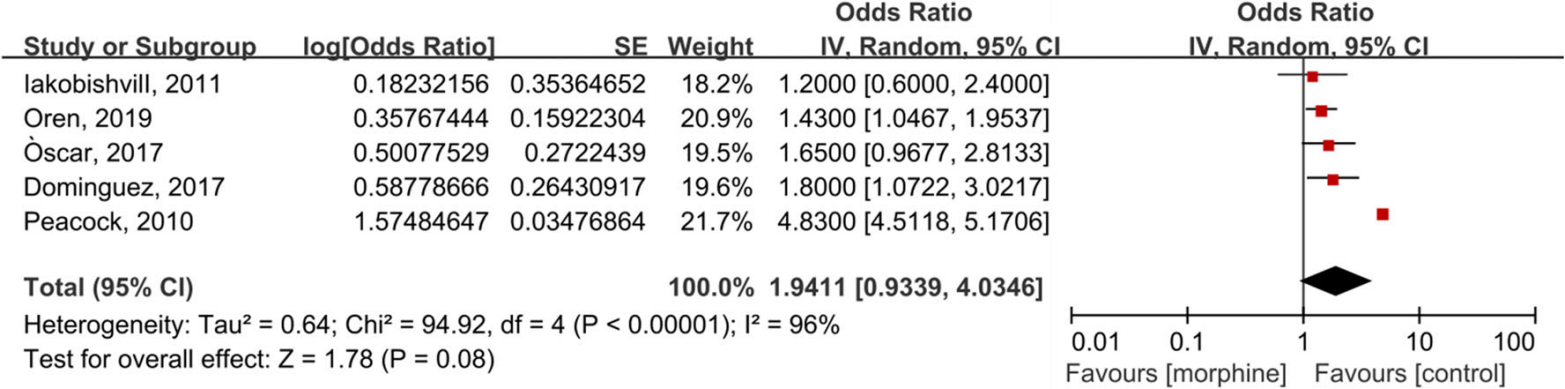
Forest plot of in‐hospital mortality

Five studies with 22 338 cases/170993 patients contributed to data for this outcome^5^, ^6^, ^14^, ^23^, ^24^ The pooled OR showed that there were no differences in in‐hospital mortality between the morphine and control groups with high heterogeneity (OR: 1.94; 95% CI 0.93 to 4.03; *p* =  0.08, I^2^ = 96%) (Figure 2).

### 3.4 The risk of invasive ventilation

**FIGURE 3:**
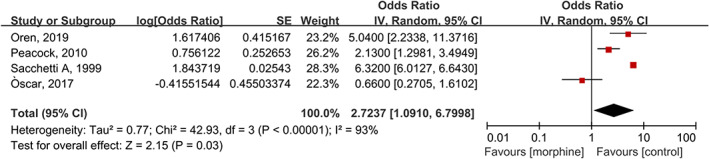
Forest plot of invasive mechanical ventilation

Four studies with 22 047 cases/167847 patients assessed the risk of invasive mechanical ventilation.^4^, ^5^, ^14^, ^24^ The results showed that morphine treatment was associated with an increased risk of invasive ventilation incidence (OR 2.72; 95% CI 1.09 to 6.80; *p* = 0.03, I^2^ = 93%) in patients with AHF compared with the control group (Figure 3).

### 3.5 7‐day and 30‐day all‐cause mortality

**FIGURE 4:**
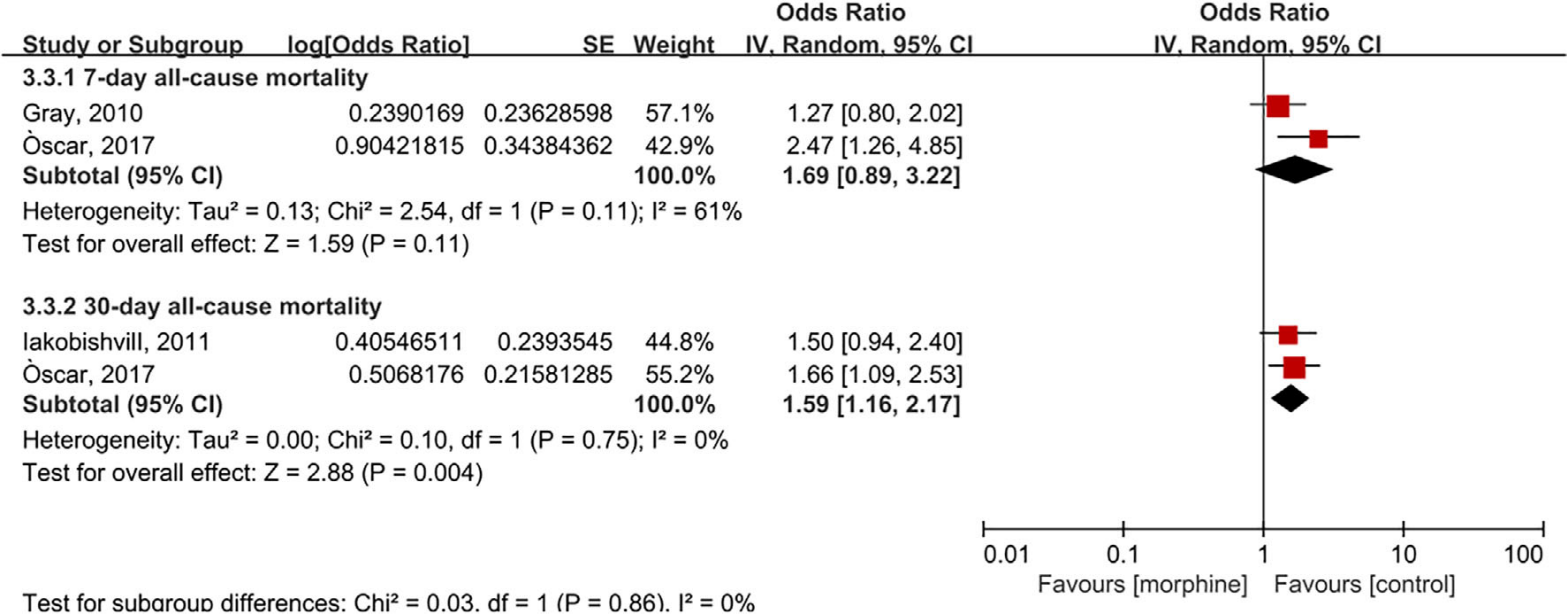
Forest plot of all‐cause mortality

Three studies with 1175 cases/9904 patients reported the association between morphine therapy and all‐cause mortality in patients with AHF.^6^, ^7^, ^14^ Subgroup analysis of all‐cause mortality was performed according to different follow‐up times between the morphine and control groups. As shown in Figure 4, the summary OR showed that there was no difference in 7‐day all‐cause mortality (OR: 1.69; 95% CI 0.89 to 3.22; *p* = 0.11, I^2^ = 61%) between the morphine and control groups. However, morphine therapy was associated with significant 30‐day all‐cause mortality (OR: 1.59; 95% CI 1.16 to 2.17; *p* =  0.004, I^2^ = 0%) with no evidence of heterogeneity.

### 3.6 Publication bias

Publication bias was not performed in this study, as the publication bias could not be ascertained, as the number of studies included for each item was <10.^25^

## 4 DISCUSSION

To the best of our knowledge, this is the first meta‐analysis to evaluate the clinical outcomes of morphine in patients with AHF. The significant findings can be summarized as follows: (1) there was no difference in in‐hospital mortality or 7‐day death between the morphine and control groups in patients with AHF; (2) morphine was associated with a higher risk of invasive ventilation and long‐term all‐cause mortality in patients with AHF.

Although morphine is used for patients with AHF based on its beneficial effects, including decreasing the preload and afterload of the heart and improving anxiety, respiratory difficulty, and chest pain,^26^ no accurate and reliable scientific data have shown the efficacy and safety of morphine for AHF. Our meta‐analysis suggests that patients with AHF showed no benefit from morphine and even had increased the risk of invasive ventilation and all‐cause mortality for long‐term follow‐up. This is basically consistent with previous studies. Daniele Orso et al.,^8^ Stefan Agewall et al., ^9^ and Kotaro Naito1 et al.^27^ reviewed the relevant research and advised that morphine should be used cautiously, or not at all, in patients with AHF.

The association between morphine and the high risk of invasive ventilation possesses several potential pathophysiological mechanisms. Excessive depression of respiratory function may be a crucial cause of induction of invasive ventilation. The negative effect of respiratory depression was also emphasized in past studies, which possibly led to intubation and ventilator therapy.^9^ Another important mechanism might be the hypotension induced by hemodynamic change.

Moreover, we also studied the role of the follow‐up time in the present meta‐analysis. We found that morphine was associated with 30‐day all‐cause mortality but not short‐term death (7‐day all‐cause mortality and in‐hospital mortality). This result should be treated with caution. The reason that morphine did not increase short‐term mortality might be that patients with AHF received multidrug therapy to relieve AHF during the 7 days after admission, which obscured the side effects of morphine to some extent. Moreover, comorbidities and drug interactions had a greater impact on short‐term mortality than on long‐term mortality, which could not reflect exactly the real pharmacological effect. Furthermore, we carefully analyzed the two articles, which included long‐term mortality. we speculated that the possible cause includes the prolonged adverse cardiovascular effect, modulation of receptor sensitivity, respiratory drive inhibition, or chronic negative inotropic action. However, only two studies were included in each subgroup analysis, which might bias the summary results. Given the above, the 7‐day all‐cause mortality might be close to in‐hospital mortality to some extent, and thus extending the follow‐up time of patients with AHF is essential.

Another point worth discussing is that the safety of morphine in patients with AHF was still controversial in different studies after propensity score matching. Oscar et al. concluded that morphine increased all‐cause mortality at different times in the EAHFE registry analysis, which conflicted with negative results given by the Israeli HFSIS registry^6^ and 3CPO trial.^7^ This might occur because the former analyzed all 46 baseline study variables, and the propensity score was ultimately calculated using 24 significant baseline variables, which was significantly more than the number of variable factors in the latter two studies. In addition, the number of patients included in the previous study was much greater. Therefore, the propensity score method cannot substitute for a prospective RTC. Future RCTs should be conducted to observe whether patients with AHF should receive morphine therapy.

However, these risks do not mean that morphine cannot improve patients' symptoms and subjective perception. As mentioned earlier, the mechanism by which morphine improves AHF symptoms includes inhibition of sympathetic nerve activity, sedative and anxiolytic effects, and improvement of lung ventilation. Therefore, it is more plausible that potential pharmacological effects overshadow the benefits for patients with AHF. The aim is to comprehensively assess the balance between risks and improvement of the symptoms through long‐term follow‐up. It is more reasonable for us to evaluate the effect of morphine for patients with AHF more comprehensively in this way.

Of course, we must point out again that our results are consistent with the current guideline recommendations, categorized as IIb or at the level of evidence (B). The evidence collected likely would not suffice to support the safety of morphine in the treatment of patients with AHF. The latest studies found that both the deterioration of renal function and the incidence of cardiogenic shock in patients with AHF are related to the use of morphine.^28^, ^29^ Undeniably, more RCTs should be conducted to observe whether patients with AHF should receive morphine. It is only with RCTs with adequate sample sizes that we will be able to provide a reliable answer to the question as to whether the use of morphine is necessary. Consequently, indications of the Guidelines of the European Society of Cardiology^12^ and the American Heart Association/American College of Cardiology^11^ are likely to stand until more extensive scientific data becomes available.

To date, no RCTs have assessed the effect of morphine on in‐hospital mortality or all‐cause mortality in patients with AHF. However, we noted that there is one ongoing multicenter and randomized control trial (MIMO trial) that aims to assess the efficacy and safety of morphine,^23^ which will not only fill our gaps in knowledge on the adverse effects and risks associated with morphine but also help to guide clinical decisions regarding the use of morphine in patients with AHF. Midazolam not only has been shown to be an efficient anxiolytic but also has positive cardiovascular effects.^30^ More specifically, compared with morphine, midazolam has sedative effects by allosterically increasing the affinity of GABAA receptors for GABA without serious side effects such as nausea, vomiting, and hypotension.^15^ Therefore, more importantly, the safety and effectiveness of another drug, midazolam, will be evaluated, which may be a viable alternative treatment.^23^

### 4.1 Study limitations

The quality of the studies included in this review was acceptable according to the quality assessment. However, the present meta‐analysis has several study limitations. First, the number included in this meta‐analysis was limited, and all the studies included were observational studies, which cannot adequately prove the causation between morphine and high mortality and the incidence of mechanical ventilation. The findings, therefore, probably have been influenced by selection bias or another residual confounding factor. Despite studies attempting to use propensity score matching to minimize selection bias, residual confounding can never be fully excluded. Performing regression analyses to account for these confounders. However, meta‐regression is no recommended by the guideline when the included studies are limited (*N* < 10). Therefore, we did not perform a meta‐regression in the present study.

It is worth noting that propensity score matching inevitably greatly reduced the sample size. As Caspi et al.^31^ mentioned before, propensity score matching has loose generalizability and precision, with potentially numerous unmeasured confounding variables. More importantly, the difference in physical status between the two groups significantly influenced the results of the study, especially in patients with AHF. Morphine‐treated patients generally represent a cohort with more severe illnesses, and they may be predicted to have greater mortality. This was not taken into account in previous studies, such as the ADHERE analysis by Peacock et al.^5^

Second, most studies did not provide a specific description of morphine therapy, such as drug dose and administration route. Therefore, due to data restrictions, we could not further analyze the effects of effective dosage or the different administration approaches of morphine in patients with AHF. Only Caspi O et al.^24^ investigated the effects of administered amounts and found that there was a significant linear dependency between the incidence of invasive mechanical ventilation and the dose of morphine.

Third, the follow‐up time of the included studies was short. Last, it is difficult to evaluate the potential pharmacological effect in vasodilatation and dyspnea amelioration of morphine owing to a lack of data regarding the degree of difficulty in breathing and changes in blood pressure. In summary, the comprehensive effects of morphine on patients with AHF need to be confirmed by more clinical information.

## 5 CONCLUSIONS

Morphine application could be associated with the increased incidence of invasive ventilation and 30‐day all‐cause mortality in patients with AHF. Future RCTs are warranted to further assess whether patients with AHF should receive morphine therapy.

## CONFLICT OF INTEREST

All the authors declared that they have no conflicts of interest.

## AUTHORS' CONTRIBUTIONS

Kui Hong was responsible for the entire project and revised the draft. Dandan Zhang and Wei Lai performed the systematic literature review and drafted the first version of the manuscript. Yang Shen and Xiao Liu reviewed, interpreted, and checked data. All authors took part in the interpretation of the results and prepared the final version of the manuscript. This work is supported by the Postgraduate Innovation Foundation of Jiangxi Province (YC2017‐B02, Wei Lai).

## Supporting information

**Table S1** Quality assessment of included studies

**Table S2** Checklist

## Data Availability

All data generated or analyzed during this study are included in this published article.
