# Supplementary material for: The safety of morphine in patients with acute heart failure: A systematic review and meta‐analysis
Source: Clin Cardiol. 2021 Jul 8;44(9):1216–24. doi: 10.1002/clc.23691 (PMC8428010; doi:10.1002/clc.23691)
Supplement: Supplementary file 1 — Table S1 Quality assessment of included studies [file CLC-44-1216-s002.doc]

**Table S1. Quality assessment of included studies**

| **Author**  **(Publication Year)** | **Newcastle-Ottawa Scale** | | | | | | | | | |  |
| --- | --- | --- | --- | --- | --- | --- | --- | --- | --- | --- | --- |
|  | **Selection** | | | **Comparability** | | | **Outcome** | | | **Total** | **Average** |
|  | **a** | **b** | **c** | **d** | **e** | **f** | **g** | **h** | **i** |  |  |
| Sacchetti *et al*, 1999 ^5^ | * | * | * |  | * | * | * |  |  | 6 | 6.43 |
| Peacock *et al,* 2010 ^6^ | * | * | * | * |  |  | * | * |  | 6 |  |
| Gray *et al*, 2010 ^8^ | * | * | * | * |  | * | * | * |  | 7 |  |
| Iakobishviliz *et al*, 2011 ^7^ | * | * | * |  |  | * | * | * |  | 6 |  |
| Oscar *et al*, 2017 ^15^ | * | * | * |  | * | * | * | * |  | 7 |  |
| Dominguez *et al*, 2017 ^23^ | * | * | * |  | * | * | * |  |  | 6 |  |
| Oren *et al*, 2019 ^24^ | * | * | * |  | * | * | * | * |  | 7 |  |

a. Representativeness of the exposed cohort.

b. Selection of the non-exposed cohort.

c. Ascertainment of exposure.

d. Demonstration that the outcome of interest was not present at the start of the study.

e. Comparability of cohorts based on the design or analysis (adjusted for age).

f. Comparability of cohorts based on the design or analysis (adjusted for any other factor).

g. Assessment of the outcome.

h. Was follow-up long enough for outcomes to occur (≥7 days).

i. Adequacy of follow-up of cohorts.
